# Social and Emotional Learning and Ninth-Grade Students’ Academic Achievement

**DOI:** 10.3390/jintelligence11090185

**Published:** 2023-09-19

**Authors:** Jessica L. Atkins, Teresa Vega-Uriostegui, Daniel Norwood, Maria Adamuti-Trache

**Affiliations:** The Department of Educational Research and Policy Studies, University of Texas at Arlington, Arlington, TX 76019, USA; teresa.hernandez3@mavs.uta.edu (T.V.-U.); daniel.norwood@mavs.uta.edu (D.N.); mtrache@uta.edu (M.A.-T.)

**Keywords:** social–emotional learning, relationship skills, school climate, academic achievement

## Abstract

A central component of adolescents’ social and emotional learning (SEL) consists of their ability to foster positive relationship skills through connectedness with their school community. This study focuses on the assessment of student’s SEL competencies in relation to their socio-demographic characteristics, formal and informal socialization behaviors, and academic outcomes in both public and private schools. The research is based on the secondary analysis of large-scale nationally representative data from the High School Longitudinal Study of 2009 (HSLS:2009) and focuses on ninth graders experiencing the transition to secondary education. Guided by both SEL and school climate frameworks, we identified survey items that describe students’ feelings of acceptance, pride, and support in their grade nine learning environment as indicators of perceptions of school climate and builders of SEL skills and used multivariate statistical analysis to examine how SEL skills and behavioral socialization affect school achievement. Study findings should inform school practitioners in developing academic and socio-cultural programs that incorporate SEL skills development.

## 1. Introduction

Educators’ awareness and interest in students’ social and emotional learning (SEL) has grown significantly over the past two decades (Eklund et al. 2018; Schonert-Reichl 2019; Williams and Jagers 2020). This also coincides with an increase in depression and suicidal ideation found among youth between the years 2009 and 2019 (Center for Disease Control 2019). As many students face obstacles to receiving emotional and behavioral care in private or community settings, schools are often expected to play a critical role in meeting these needs (Durlak et al. 2011; Farmer et al. 2003). With a significant number of students experiencing social–emotional difficulties and a need for students to be socially and emotionally competent and prepared for their educational journeys, educators must be cognizant of the impacts of emotional regulation and social skills on academic achievement. For instance, according to Maslow’s Hierarchy of Needs (Maslow 1954), a child’s well-being and sense of belonging must be adequately addressed before the child can effectively learn, apply knowledge, and solve problems. Therefore, a holistic approach to development, including social and emotional competencies, is a crucial factor in students’ academic success. In addition, educators must be trained to integrate the development of SEL skills in the classroom curriculum, while school leaders must focus on building a schoolwide SEL by improving school climate, policies, and practices and engaging in family and community partnerships (Oberle et al. 2016). Research shows that school climate is more than an organizational attribute (Anderson 1986) because students’ perceptions of a safe and supportive climate affect the development of their social and emotional learning skills (Osher and Berg 2017; Zelinski and Villenas 2020). However, in most schools, especially public schools, educators have limited time and financial resources available to balance academic performance goals with those of social–emotional development (Durlak et al. 2011). 

### 1.1. Social–Emotional Learning

In recent years, social–emotional learning (SEL) practices have become a priority in many schools across the globe (Tan et al. 2018; Taylor et al. 2017; Yang et al. 2020). The Collaborative for Academic, Social, and Emotional Learning (CASEL) defines SEL as a process of teaching children, adolescents, and adults the knowledge and skills necessary to develop self-awareness, manage emotions, gain empathy toward others, develop and maintain relationships, and make decisions that are healthy for oneself and others (CASEL 2022). These skills are encompassed by five competencies: self-awareness, self-regulation, social awareness, relationship skills, and responsible decision making (CASEL 2022). Numerous evidence-based programs have been designed to foster these skills in students, with the goal of teaching emotional regulation and interpersonal skills needed to work effectively with others (Ahmed et al. 2020; Denham et al. 2012; Eklund et al. 2018; Schonert-Reichl 2019). SEL programs have been developed to address a wide range of behavioral, social, and learning needs, from prevention to intervention (Eklund et al. 2018). 

An important area of research in SEL is the potential impact of these programs on mental health outcomes. Studies have shown that SEL programs may reduce students’ feelings of anxiety and depression, improve self-esteem, and enhance overall well-being (Greenberg et al. 2003). There is also evidence to suggest that SEL can be particularly beneficial for students who have experienced trauma or adversity (Gloppen et al. 2018). Additionally, research has indicated that SEL programs can result in positive school-level outcomes such as improved school climate and reductions in disciplinary issues (Walton et al. 2022).

Evidence also suggests that SEL programs are cost-effective, with an eleven-dollar benefit per dollar spent on the program (Belfield et al. 2015; Eklund et al. 2018). Mental health treatment in clinical settings tends to be costly, which can often be a barrier for low-income populations (Jones et al. 2010). Therefore, the preventative nature of SEL programs in schools may be particularly beneficial for these populations. The cost-effectiveness of SEL programs is also reflected in positive outcomes for students, including increased academic engagement, positive relationships, emotional regulation, and a sense of belonging in the school community (Eklund et al. 2018; Schonert-Reichl 2019). Furthermore, the cost–benefit of SEL programs was also reflected in a decrease in adverse outcomes, such as disciplinary or special education referrals due to behavioral disorders difficulties (Eklund et al. 2018). 

### 1.2. Relationship Skills

One of the five SEL competencies is relationship skills, which is defined as an individual’s ability to build and maintain positive, supportive relationships; communicate effectively; resolve conflicts; seek and offer support; and navigate differences in social settings (CASEL 2022). Research has indicated that interpersonal relationships are important for students’ intellectual and academic development (Bowlby 1969; Cemalcilar 2010; Furrer and Skinner 2003). Schools play an important role in providing students with opportunities to engage with others and develop relationships outside of the family (Cemalcilar 2010; Keppens and Spruyt 2019; Walton and Brady 2020). Developing student social relationships and school connectedness has major effects on building healthy school climates, as demonstrated by Zullig et al. (2010).

Quality relationships with teachers also contribute to student motivation and positive attitudes about school (Allen et al. 2021; Cemalcilar 2010; Furrer and Skinner 2003; Hoffmann et al. 2023; Hughes and Kwok 2007) and are associated with positive academic outcomes (Roeser et al. 1996). Quality relationships appear to be particularly important for vulnerable or at-risk students as these relationships foster a sense of safety and belonging in school (Sanders and Munford 2016). Nurturing students’ sense of belonging at school (SOBAS) has received worldwide attention among educators because it is recognized as central to students’ psychosocial well-being and academic success (Chiu et al. 2016). However, there is also evidence that suggests that the effectiveness of school-based intervention programs, including SEL, may be affected by individual characteristics of students and the characteristics of schools (Hughes et al. 2005). For example, prior research has indicated that students who identify as female tend to report higher climate scores for their schools than students who identify as male (Keppens and Spruyt 2019; Koth et al. 2008; White et al. 2014; Xu et al. 2021).

Additionally, the effectiveness of SEL programs may vary between schools that serve large percentages of low SES students versus those that serve lower percentages of low SES students (Hughes et al. 2005). Differences in academic achievement between private and public schools may also be explained by better school climate in private schools (Dronkers and Robert 2008; Shakeel and DeAngelis 2018). A growing body of empirical research supports the idea that both SEL practices and school climate reform have a positive effect on academic learning, so “educating the whole child” begins with a positive school climate (Cohen 2017; Darling-Hammond and Cook-Harvey 2018). Some argue that school climate may have a moderating role between the schoolwide SEL and student outcomes such as cyberbullying victimization (Yang et al. 2020), which means student SEL perceptions may be associated with the school setting. Additionally, prior research has indicated that a positive school climate may have clear social norms, thereby encouraging prosocial behaviors and responsible decision making among students, which then leads to stronger SEL competencies (Pan et al. 2023). Pan et al. also argued that school climate might serve as a foundation for students’ development of social–emotional skills by providing a supportive and safe environment for learning. Similarly, Zelinski and Villenas (2020) describe the symbiotic relationship between SEL and school climate. This assertion is in line with other studies that reflect the impact of social connections on SEL competencies (La Greca and Harrison 2005; Roeser et al. 2000).

While social–emotional skills and academic achievement have been historically viewed as mutually exclusive sets of skills, research has demonstrated that children’s ability to regulate their emotions and make healthy connections with others contribute to their academic learning (Durlak et al. 2011; Lemberger-Truelove et al. 2021; Walton et al. 2022). Specifically, research has indicated that SEL programs can yield improvements in achievement as measured by tests and grades (Durlak et al. 2011). Additionally, there is evidence to suggest that SEL can have long-term benefits, such as increased graduation rates and higher rates of college attendance (Jones et al. 2015). This may be explained by SEL programs assisting students in gaining prosocial abilities (e.g., sustaining healthy relationships, regulating stress, recovering from setbacks, and making responsive decisions), which then serve as protective factors that mitigate maladaptive or risky behaviors that could impede academic outcomes (Panayiotou et al. 2019). This is further supported by evidence that mental health, social behaviors, and academic achievement are highly intertwined, particularly as children spend more time in school than previous generations did and are also gaining more exposure to issues of mental health and social isolation (Cristovao et al. 2017; Panayiotou et al. 2019). However, some researchers have questioned the relationship between SEL and academic achievement (Duncan et al. 2007; Zeidner et al. 2002). Nevertheless, other researchers have provided evidence of a relationship between SEL competencies and academic achievement, including relationship skills (Walton et al. 2022).

### 1.3. Current Study

While SEL and its impact on student behavior and achievement have gained increasing focus over the past decade, existing research has tended to focus on early childhood and elementary-age students, with limited attention to secondary-level students. Therefore, we aimed to address this gap by utilizing data from the High School Longitudinal Study of 2009 (HSLS:2009) that follows ninth graders’ educational journey during high school years and beyond. Specifically, we focused on exploring whether informal socialization (with school staff, peers, and family) and formal socialization (participation in official school groups that build social capital) are correlated with students’ relationship skills and whether relationship skills reported by students and behavioral socialization are related to their academic achievement. The current study uses the terms SEL relationship skills and perceptions of school climate interchangeably because once students perceive the school climate as supportive, they are likely to have developed social and emotional skills. 

Furthermore, the study is based on the assumption that school culture may affect the development of SEL relationship skills, which has indirect consequences on student achievement. School culture is defined as shared behaviors, values, and relationships between individuals within the school (Cakiroglu et al. 2012), which is likely different in public and private schools. We hypothesized that both formal and informal socialization opportunities and school culture differences in the public and private sectors would be factors affecting students’ relationship skills and, as a result, academic achievement. Nevertheless, we anticipate that students’ socio-demographic characteristics, including family background, may also affect the relationship between relationship skills and academic achievement.

Therefore, the purpose of this study is to examine the complex relationship between student SEL relationship skills, their socio-demographic characteristics, formal and informal socialization behaviors, and academic outcomes in public and private school environments. For this research, SEL competencies are described by students’ feelings of acceptance, pride, and support in their learning environment as indicators of relationship-building skills within the school community. The study addresses the following research questions:

1. Are there any differences in SEL relationship skills scores by student socio-demographic characteristics (i.e., sex, race/ethnicity, parental education, SES) and formal and informal socialization behaviors (i.e., participation in college access programs, relationships) when comparing public and private schools?
2. To what extent do SEL relationship skills scores and formal and informal socialization behaviors affect school achievement when controlling for student socio-demographic characteristics and type of school?

## 2. Materials and Methods

This is a secondary analysis of public-use nationally representative data from the High School Longitudinal Study of 2009 (HSLS:2009) available through the National Center for Education Statistics (Ingels et al. 2011). HSLS: 2009 data follow over 23,000 9th graders from 944 schools surveyed throughout their secondary and postsecondary years between ages 15 and 22. The current study used only the 2009 survey data when participants were in their 9th grade school year. The final research sample consists of *n* = 17,542 students from public and private schools with valid information on all study variables.

### 2.1. Variables

Understanding the relationship between students’ social–emotional feelings and socialization behaviors with academic achievement was foundational to this study. Academic achievement is the main dependent variable defined as the grade 9 GPA, a continuous measure varying from 0 to 4.

Relationship skills are hypothesized to be a significant determinant of achievement, also explored in relation to student socialization factors and school factors that contribute to its development. The HSLS data include survey items that describe students’ perceptions of their social–emotional well-being at school (e.g., feelings of safety, pride, and connectedness within the school) and build on the notion of student’s sense of belonging at school. These survey items measured on a 4-point Likert scale (1 = Strongly Disagree; 2 = Disagree; 3 = Agree; 4 = Strongly Agree) fit into a single component that we describe as a measure of relationship skills (Appendix A, Table A1). The relationship skills scale consists of 3 items, and Cronbach’s alpha coefficient of 0.714 indicates a good scale reliability, so a mean relationship skills score was derived.

For the purpose of this study, other survey items were selected to measure the socialization behaviors of students engaging in discussions about their personal problems with parents, with teachers or school counselors, and with friends, which indicates a desire to build social relationships (see Table A1). Three dichotomous variables were introduced as indicators of informal socialization behaviors. Moreover, participation in school programs (e.g., Talent Search, Upward Bound, Gear Up, AVID, MESA) is also known as academic and social capital builders, so student participation in at least one program is proposed as an indicator of formal socialization behavior.

Finally, we argue that public and private schools may foster different school cultures embraced by students, school personnel, and parents that may impact both the development of social connections and, ultimately, academic achievement. We also selected student socio-demographic characteristics as control variables: gender (2-category variable), race/ethnicity (6-category variable), parental education (4-category variable), and socioeconomic status quintiles (treated as ordinal variable in the regression models).

### 2.2. Statistical Procedures

Preliminary exploration of HSLS data showed significant differences in the socialization factors by school type. We used frequency distributions and descriptive statistics to describe the sample and compare the relationship skills scores by school type. We conducted 2-way ANOVA tests to compare relationship skills mean scores by school type and study variables. We also developed separate sequential regression models for public and private schools. All analyses used normalized weights based on the base year student analytic weight (W1STUDENT) to ensure a correct estimation of population proportions while reporting counts of the research sample.

## 3. Results

### 3.1. Research Sample

The research sample represents about 75% of the HSLS nationally representative sample and includes cases with complete information on all study variables. Nevertheless, gender, racial, and socioeconomic distributions are not significantly altered in the research sample compared to the HSLS nationally representative sample.

Table 1 presents the student characteristics, socialization factors, and student outcomes for the entire research sample and for the public and private school subsamples. Private school students represent about 7% of the entire sample, and this group includes larger percentages of female and White students coming from more educated and affluent family backgrounds. These students are more likely to share their personal problems with school personnel, parents, and friends but less likely to participate in formal socialization programs that may not even be available in the private school sector. They also score higher on the relationship skills and academic achievement measures. Public and private schools have different student compositions and different levels of socialization that are reflected in the social and academic development of students.

### 3.2. SEL Relationship Skills Scores

As previously discussed, the SEL relationship skills measure is a composite score of selected survey items that describe the student’s sense of belonging within their school community. To address the first research question, we conducted eight two-way ANOVA analyses to identify the main effects and interactions when comparing relationship skills scores by school type and each study variable (i.e., sex, race/ethnicity, parental education, SES, and four socialization factors). First, Table 2 shows the means and standard deviations of relationship skills scores for each category of the study variables by school type. Cohen’s d effect size indexes are also included. We notice that the average relationship skills scores in the range of 3 to 3.5 indicate agreement with the statements describing student comfort and connectedness with school, although clearly, students in private schools have higher relationship skills scores. However, Cohen’s d effect sizes varying between 0.4 and 0.8 indicate only medium practical significance of the mean differences.

Second, Table 3 presents the results of each of the eight two-way ANOVA tests, including main effects and interactions. All analyses indicate statistically significant (*p* < .001) main effects for school type. However, the effect size for school type was small in all two-way ANOVA analyses with partial eta squared η^2^ varying between 0.004 and 0.015. The main effects were statistically significant only for some study variables, such as parental education, SES quintiles, talking to school personnel, talking to parents, and talking to friends, although the corresponding effect sizes measured by partial eta squared η^2^ were very small (<0.001). Third, there was no interaction effect between any of the eight study variables and school type, which suggests private school students scored above public school students regardless of the socio-demographic and socialization factors.

### 3.3. Achievement Models

We conducted separate sequential linear regression models for public and private schools that examine the relative contribution of relationship skills and socialization factors on ninth graders’ school achievement while controlling for student socio-demographic characteristics. This allows us to compare which school type the model was better fit by predictors. Model 1 includes only the relationship skills scores and the socialization factors, and Model 2 includes all study variables. Table 4 presents comparatively the findings for public and private schools in terms of unstandardized coefficients and the significance of the corresponding t-tests. The adjusted R^2^ is a corrected goodness-of-fit measure for linear models and identifies the percentage of variance in the outcome that is explained by the selected independent variables. For public and private schools, Model 1 explains 6.5% and 5.8% of the variation in achievement, respectively. When controlling for student characteristics (Model 2), the adjusted R^2^ becomes 21.8% and 15.3% for public and private schools, respectively. The higher value for public schools may indicate stronger effects due to more variability in student backgrounds.

#### 3.3.1. Public School Models

Model 1: As hypothesized in the model, relationship skills measures and socialization factors have an impact on achievement. One unit increase in relationship skills scores is associated with a 0.231 increase in GPA. Some socialization factors, like talking to parents and especially to friends about personal problems, are positively related to achievement. Meanwhile, there is a negative relationship between talking to school personnel and achievement, possibly because students with academic problems were more likely to need their support. Similarly, formal socialization through school programs (e.g., Talent Search, Upward Bound, Gear Up, AVID, MESA) designed as academic and social capital builders appears to be negatively associated with achievement because they may attract low-achieving students in need of more support. Since the relationships indicated by the model are not causal, it is likely that low-achieving and high-achieving students could respond to different socialization factors.

Model 2: The full model with all factors included explains 21.8% of the variability in the outcome. Student characteristics are significant in predicting academic achievement, and they only partially change the contribution of relationship skills scores and socialization factors to the outcome. First, Model 2 maintains the direction of the relationships between each factor and achievement. Factors are statistically significant at 0.001 level, except for the formal socialization factor (i.e., participation in a social program) that is losing its effect on achievement. Second, the effect of socio-demographic factors appears to be strong for public schools. Male students have lower achievement than female students. All racial groups except Asians have lower grade nine GPAs when compared to White students. Academic achievement is higher for students whose parents have bachelor’s or graduate degrees, and one unit increase in SES quintiles corresponds to a 0.124 increase in GPA.

#### 3.3.2. Private School Models

Model 1: The direction of relationships between relationship skills measures and socialization factors and achievement is similar for private schools, although some factors do not have statistically significant effects. One unit increase in relationship skills scores is associated with a 0.121 increase in GPA, which is lower than the public school result. When compared to public schools, the factor of talking to parents appears to be more important than talking to friends about personal problems, but both factors are positively related to achievement. The relationship between the factor of talking to school personnel and GPA is not statistically significant, while the formal socialization through school programs factor is also negatively associated with achievement (and statistically significant at the 0.05 level).

Model 2: The full model with all factors included explains 15.3% of the variability in the outcome. Student characteristics are also significant predictors of academic achievement, and they slightly decrease the contribution of relationship skills scores and socialization factors to the outcome. First, the only factors statistically significant at 0.001 level are the relationship skills scores and talking to parents. The factors indicating talking to friends continue to have a significant but lower positive effect on GPA when controlling for student socio-demographic characteristics. Second, the effect of socio-demographic factors on achievement appears to be weaker for private schools. Male students and Black students have significantly lower achievement, while effects are very weak for the other racial categories. Academic achievement is higher for students whose parents have obtained any postsecondary degree, although the strongest effects on GPA are obtained for students with university-educated parents. For private schools, there is no significant effect of SES on students’ GPA, which may reflect more uniformity of family backgrounds in private schools.

## 4. Discussion

The present study sought to examine high school students’ perceptions of school climate as builders of SEL relationship skills in relation to their socio-demographic characteristics, formal and informal socialization behaviors, and the overall effect of these factors on academic outcomes in both public and private schools. By using student responses from the High School Longitudinal Study of 2009, the study provides an account of the relevance of relationship skills and behavioral socialization factors on academic achievement for a large-scale, nationally representative sample.

As hypothesized, the regression models show a positive effect of relationship skills scores on academic achievement. This result is in agreement with previous research, which has indicated that social skills and interpersonal relationships are important factors in students’ academic development (Bowlby 1969; Cemalcilar 2010; Furrer and Skinner 2003). This effect was stronger for public school students than private school students, as also predicted by prior studies that revealed differences in SEL program effectiveness based on student socio-demographics (Hughes et al. 2005) and differences in academic achievement between private and public schools due to school climate (Dronkers and Robert 2008). School climate and academic outcomes in private schools may be positively affected by smaller school sizes or smaller student-to-teacher ratios (Humlum and Smith 2015; Karakutu et al. 2014; Weiss et al. 2010). As a result, this potentially explains that while private school students included in the HSLS survey had overall lower participation in formal socialization programs, their relationship skills index scores tended to be higher than those of public school students. In addition, the household characteristics of students in private and public schools are different (Wang et al. 2019), with a lower percentage of private school students living in poor households (8% vs. 18%), almost 60% having parents with at least a bachelor’s degree compared to 40% for public school students, and over 80% coming from two-parent families compared to 65–70% of public school students. This suggests that private school students acquire more cultural, social, and economic capital and thus are more trained in social competence by possessing the ability to comply with social norms and the understanding of the school system and power relationship (Bourdieu and Passeron 1990).

The regression models also showed a positive relationship between academic achievement and student interaction with parents and friends. The positive effect of talking to parents was more pronounced for private school students while talking to friends was more effective for public school students. These effects were all present with and without control variables (students’ socio-demographic characteristics), indicating that students’ academic achievement benefitted from higher ratings of relationship skills as well as interactions with parents and friends, regardless of the student’s gender, race, socioeconomic status, and parental level of education. The importance of informal socialization on achievement was also predicted by previous studies, which found interpersonal relationships to be important factors in academic development (Bowlby 1969; Cemalcilar 2010; Furrer and Skinner 2003).

However, socio-demographic factors accounted for a significant amount of variance within the outcomes, especially for the public school student populations, which tend to be more diverse. Lower achievements were obtained by male students and by Indigenous, Black, Hispanic, and Multiracial students, while higher achievements were demonstrated by Asian students. Similar achievement patterns are also found by the National Center for Education Statistics (NCES) in their 2018 Status and Trends in the Education of Racial and Ethnic Groups report (NCES 2019). Among private school students, a significant negative effect on GPA was obtained only for Black students. Although parental education (i.e., parents with a bachelor’s degree or above) showed a positive effect on achievement for both public and private school students, the economic differences measured by family SES were only significant for the public school students. The achievement gaps related to parental education as a source of cultural capital and SES as an indicator of economic capital are not surprising and have been associated with cultural reproduction across generations through the socializing influence within educational institutions (Bourdieu and Passeron 1990).

To our surprise, the achievement models also showed that students’ interactions with school personnel and participation in school social programs were not associated with higher academic achievement. This was in contrast to other studies, which indicated that quality relationships with school personnel are associated with positive academic outcomes (Cemalcilar 2010; Furrer and Skinner 2003; Hoffmann et al. 2023; Hughes and Kwok 2007; Roeser et al. 1996). However, in our study, the negative relationship between talking to school personnel and achievement was only significant for public school students. Additionally, while participation in social programs had a negative relationship with achievement for all students, it was no longer significant when the socio-demographic variables were included. The surprising relation between achievement and talking to school personnel among public school students could be related to factors such as students who are struggling academically spending more time with teachers through tutorials or other academic interventions. However, the survey did not provide specific information about the nature or context of interactions with teachers. Therefore, further study is necessary to determine whether different contexts of interactions between students and teachers have different outcomes on academic achievement.

A key finding of the study was to reveal differences between private and public schools with respect to student composition, relationship skills, social interactions, and academic achievement. Differences in student composition are not surprising (Hughes et al. 2005), and they may explain the differences in relationship skills scores and achievement outcomes. First, students in private schools may have advantages over students in public schools in terms of possessing more cultural capital (i.e., parental education), economic capital (i.e., higher SES), and social capital (i.e., higher levels of socialization opportunities outside of school). Furthermore, a study conducted by Marks and Kuss (2001) examined socialization for citizenship differences between public and private school students. Marks stated that within the confines of private schools, there is an expectation to meet traditional values, such as community, school, and family involvement. This may result in increased opportunities for students to actively engage with the community and their peers, resulting in more exposure to activities that promote the development of relationship skills. However, while the relationship skills composite scores were higher overall for private school students compared to public school students, differences in composite scores were more pronounced among public school students who represent a more diverse population. While these differences were revealed between public and private school students, the academic achievement of both groups (as measured by ninth-grade GPA) was affected by their relationship skills composite scores. These findings are consistent with prior research, which has indicated that social–emotional competencies, including relationship skills, are associated with higher academic achievement, graduation rates, and college attendance (Durlak et al. 2011; Jones et al. 2015; Lemberger-Truelove et al. 2021; Walton et al. 2022).

## 5. Conclusions

The findings of this study affirmed the importance of formal and informal socialization in developing students’ sense of connectedness in the school community, as well as the positive effects these factors can have on academic achievement. As previous research has argued the interconnectedness of SEL and school climate (La Greca and Harrison 2005; Roeser et al. 2000; Pan et al. 2023; Yang et al. 2020), our findings reflect the importance of SEL in building a climate that promotes relationship skills and positive academic outcomes. Therefore, continued efforts related to social–emotional learning policies and procedures will likely be beneficial to students by addressing the social and emotional competencies needed to succeed in school and beyond.

Additionally, study findings revealed differences between public and private school students’ feelings of connectedness and belonging in their school community, with students in private schools having higher relationship skills scores. While this may be explained by private school students having individual advantages in securing various forms of capital, future research should explore any other socialization factors in the private school environment that may be contributing to these differences. For example, private schools may tend to have smaller student-to-teacher ratios or resources that are not available in most public schools. Further attention to these differences may provide practical implications for policy and practice, particularly if any gaps are discovered in public school structures or practices.

Furthermore, the findings of this study support a need for future research into the influence of other socialization opportunities, such as participation in athletics, band, or theater. The formal socialization factors included in the HSLS 2009 survey consisted of college access programs, through which students may be more focused on their individual plans and goals than the aforementioned activities, which are more focused on collective goals. However, the survey did not include questions about students’ participation in team-oriented activities or clubs. Future research should explore whether or not the cooperative, collaborative nature of these activities may exert a stronger influence on developing students’ relationship skills that, in turn, could affect their academic achievement.

## Figures and Tables

**Table 1:** Descriptive statistics of research sample by school type (column % or means scores).

	All (N = 17,542)	Public (N = 16,322)	Private (N = 1220)
Socio-demographic characteristics			
Sex			
Female	50.9	50.7	53.4
Male	49.1	49.3	46.6
Race/ethnicity			
Indigenous	1.1	1.1	1.3
Asian	3.6	3.5	3.7
Black	12.4	12.9	5.8
Hispanic	21.9	22.7	12.1
Multiracial	7.8	7.9	6.6
White	53.2	51.9	70.4
Parental education			
High school/less	46.3	48.5	17.0
Associate degree	16.5	17.0	9.6
Bachelor’s degree	22.0	20.9	37.3
Graduate degree	15.3	13.7	36.2
SES mean score (range 1–5)	3.06	2.97	4.24
Socialization factors (% Yes)			
Talk to school personnel	14.4	14.3	15.7
Talk to parents	61.9	61.3	69.4
Talk to friends	61.8	61.5	66.0
Participation in social programs	12.2	12.6	7.4
Student outcomes			
Relationship skills mean scores (range 1–4)	3.13	3.11	3.44
Average grade 9 GPA (range 0–4)	2.66	2.62	3.15

**Table 2:** Means (SDs) for SEL relationship skills scores by school type and study variables.

	School Type		Cohen’s d
	Public Schools	Private Schools	
Socio-demographic characteristics			
Sex			
Female	3.11 (0.54)	3.44 (0.49)	0.60
Male	3.11 (0.60)	3.44 (0.52)	0.56
Race/ethnicity			
Indigenous	3.08 (0.46)	3.31 (0.63)	0.49
Asian	3.05 (0.60)	3.30 (0.52)	0.41
Black	3.09 (0.61)	3.43 (0.64)	0.55
Hispanic	3.06 (0.54)	3.48 (0.46)	0.77
Multiracial	3.02 (0.62)	3.49 (0.51)	0.76
White	3.15 (0.56)	3.44 (0.50)	0.51
Parental education			
High school/less	3.08 (0.58)	3.43 (0.48)	0.61
Associate degree	3.09 (0.58)	3.40 (0.52)	0.54
Bachelor’s degree	3.16 (0.55)	3.43 (0.50)	0.51
Graduate degree	3.19 (0.55)	3.46 (0.52)	0.51
SES quintiles			
First (lowest)	3.07 (0.59)	3.35 (0.53)	0.46
Second	3.08 (0.59)	3.44 (0.54)	0.61
Third	3.07 (0.57)	3.47 (0.50)	0.70
Fourth	3.13 (0.55)	3.41 (0.47)	0.51
Fifth (highest)	3.20 (0.54)	3.45 (0.52)	0.47
Socialization factors			
Talk to school personnel			
No	3.09 (0.57)	3.43 (0.49)	0.60
Yes	3.22 (0.58)	3.50 (0.56)	0.48
Talk to parents			
No	2.97 (0.60)	3.22 (0.49)	0.59
Yes	3.20 (0.54)	3.49 (0.56)	0.55
Talk to friends			
No	3.07 (0.60)	3.42 (0.49)	0.57
Yes	3.13 (0.55)	3.45 (0.52)	0.58
Participation in social programs			
No	3.11 (0.56)	3.43 (0.51)	0.58
Yes	3.13 (0.63)	3.53 (0.46)	0.65

**Table 3:** Two-way ANOVA tests: SEL relationship skills scores by school type and study variables.

	F Statistics	*p*-Value
School type and sex		
Main effect—school type	F(1, 16,870) = 264.505	<0.001
Main effect—sex	F(1, 16,870) = 0.057	0.812
Interaction effect	F(1, 16,870) = 0.047	0.828
School type and race/ethnicity		
Main effect—school type	F(1, 16,862) = 72.493	<0.001
Main effect—race	F(5, 16,862) = 1.563	0.167
Interaction effect	F(5, 16,862) = 1.813	0.107
School type and parental education		
Main effect—school type	F(1, 16,866) = 155.239	<0.001
Main effect—parental education	F(3, 16,866) = 2.684	0.045
Interaction effect	F(1, 16,866) = 0.654	0.580
School type and SES		
Main effect—school type	F(1, 16,864) = 92.534	<0.001
Main effect—SES	F(4, 16,864) =2.638	0.032
Interaction effect	F(4, 16,864 ) = 1.255	0.285
School type and talk to school personnel		
Main effect—school type	F(1, 16,870) = 126.451	<0.001
Main effect—talk to school personnel	F(1, 16,870) = 12.783	<0.001
Interaction effect	F(1, 16,870) = 1.115	0.291
School type and talk to parents		
Main effect—school type	F(1, 16,870) = 226.217	<0.001
Main effect—talk to parents	F(1, 16,870) = 86.488	<0.001
Interaction effect	F(1, 16,870) = 1.976	0.160
School type and talk to friends		
Main effect—school type	F(1, 16,870) = 235.109	<0.001
Main effect—talk to friends	F(3, 16,870) = 5.022	0.025
Interaction effect	F(1, 16,870) = 0.340	0.560
School type and participation in social programs		
Main effect—school type	F(1, 16,870) = 93.620	<0.001
Main effect—participation in social programs	F(1, 16,870) = 2.479	0.115
Interaction effect	F(1, 16,870) = 1.054	0.305

**Table 4:** Sequential linear regression models of academic achievement for public and private schools.

	Public Schools		Private Schools	
	Model 1	Model 2	Model 1	Model 2
SEL relationship skills scores	0.231 ***	0.187 ***	0.121 ***	0.113 ***
Talk to school personnel (No = ref)				
Yes	−0.209 ***	−0.171 ***	−0.050	−0.043
Talk to parents (No = ref)				
Yes	0.183 ***	0.142 ***	0.247 ***	0.199 ***
Talk to friends (No = ref)				
Yes	0.269 ***	0.130 ***	0.174 ***	0.079 *
Participation in social programs (No = ref)				
Yes	−0.154 ***	−0.034 ^+^	−0.142 *	−0.095
Sex (Female = ref)				
Male		−0.230 ***		−0.276 ***
Race/ethnicity (White = ref)				
Indigenous		−0.376 ***		−0.222
Asian		0.357 ***		0.161 ^+^
Black		−0.414 ***		−0.329 ***
Hispanic		−0.319 ***		−0.097 ^+^
Multiracial		−0.216 ***		0.011
Parental education (High school = ref)				
Associate degree		−0.013		0.141 ^+^
Bachelor’s degree		0.136 ***		0.189 **
Graduate degree		0.209 ***		0.337 ***
SES		0.124 ***		0.027
Constant	1.677 ***	1.727 ***	2.469 ***	2.422 ***
Adjusted R^2^	6.5%	21.8%	5.8%	15.3%

*** *p* < .001; ** *p* < .01; * *p* < .05; ^+^ *p* < .1.

## Data Availability

The data presented in this study are available from the National Center for Education Statistics (NCES) at https://nces.ed.gov/surveys/hsls09/ (accessed on 10 January 2023).

## Appendix A

**Table A1:** HSLS survey items.

Variable Name	Variable Description
SEL Relationship Skills items	
S1SAFE	S1 E01A 9th grader feels safe at school
S1PROUD	S1 E01B 9th grader is proud to be part of his/her school
S1TALKPROB	S1 E01C 9th grader has teacher/adult in school he/she can talk to about problems
Socialization items	
S1MOMTALKPRB & S1DADTALKPRB	S1 E11A/B 9th grader talked to mother (& to father) about personal problems
S1FRNDTLKPRB	S1 E11C 9th grader talked to friends about personal problems
S1TCHTALKPRB & S1CNSLTLKPRB	S1 E11D/E 9th grader talked to teacher (& to school counselor) about personal problems
S1TALENTSRCH & S1UPWARDBND & S1GEARUP & S1AVID & S1MESA	S1 E16A-E 9th grader is participating in Talent Search, Upward Bound, Gear Up, AVID or MESA
